# Levels, Trends and Disparities in Public-Health-Related Indicators among Reproductive-Age Women in Bangladesh by Urban-Rural and Richest-Poorest Groups, 1993-2011

**DOI:** 10.1371/journal.pone.0075261

**Published:** 2013-09-25

**Authors:** Md. Mobarak Hossain Khan, Arina Zanuzdana, Alexander Kraemer

**Affiliations:** Department of Public Health Medicine, School of Public Health, University of Bielefeld, Bielefeld, Germany; University of New South Wales, Australia

## Abstract

**Background And Objectives:**

Although Bangladesh has already achieved noticeable progress in the field of development and health, disparities in public health indicators for several markers are still reported. To assess public health development in Bangladesh during the last two decades, firstly, we analysed levels, trends and disparities in public-health-related indicators by rural versus urban as well as by the richest versus poorest group of women who have ever been married. Secondly, using the most recent data set we performed multiple analyses to check whether urban-rural and richest-poorest disparities were still significant.

**Methods:**

The analysis was based on six nationally representative data sets from the Bangladesh Demographic and Health Surveys (BDHS) conducted in 1993-94 (n=9,640), 1996-1997 (n=9,127), 1999-2000 (n=10,544), 2004 (n=11,440), 2007 (n=10,996) and 2011 (n=17,749). The outcome variables were six selected public-health-related indicators. We performed various types of analyses, including multiple logistic regressions.

**Results:**

The trend of all indicators except being overweight (1993-2011) displayed gradual improvements for both markers. However, the urban and richest groups revealed a better situation than their counterparts in both simple and multiple analyses. Disparities between richest-poorest groups were more pronounced than urban-rural disparities. For instance, the prevalence of delivery at any healthcare facility in 2011 was 20.4% in rural areas and 46.5% in urban areas, whereas it was 9.1% in the poorest group and 57.6% in the richest group.

**Conclusion:**

The public health sector in Bangladesh has achieved some successes over the last two decades. However, urban-rural and richest-poorest disparities are still considerable and therefore more public health strategies and efforts are clearly needed for the rural and poorest groups of women in order to reduce these gaps further.

## Introduction

Bangladesh has achieved noticeable progress since its independence in 1971 despite many constraints like environmental disasters, rapid population growth and limited resources. The declining trends of poverty, illiteracy and infant, child and maternal mortality, as well as increasing life expectancy are a few examples of achievement [1-4]. Infant mortality per 1,000 live births declined from 87 in 1993-94 to 43 in 2007-2011. Even more impressive achievements have been observed for post-neonatal and under-five mortality. In particular, the rate of decline was faster in rural than urban areas, which reduces the gaps in child mortality significantly [5]. Some of the important factors that might have contributed to this development are the ratification and implementation of many international treaties and declarations, an increasing national commitment to promoting institutional capacity and upholding civil rights, greater emphasis on female education, extending healthcare systems throughout the country, the implementation of micro-credit income generating programmes for the poor, the introduction of an old-age pension for this vulnerable group, timely implementation of suitable public-health interventions (e.g. childhood vaccination) and steadily rising economic expansion through industrialisation and foreign remittances [1,3,6-11].

Although the above-mentioned achievements and programmes are praiseworthy, Bangladesh still needs a lot of effort from both governmental and non-governmental organisations (NGOs) to reduce e.g. poverty and health disparities. Nationally, about 40% of the total population lives in poverty [3], which is considerably higher in rural than urban areas [1]. The urban-rural disparity in terms of healthcare is also significant. Rural people have limited access to facilities to receive healthcare from trained personnel and specialised hospitals [6]. The healthcare system is generally biased towards the rich and urban elites [3,6,10]. In general, economically sound families, males and urban residents are more privileged in terms of admission and they receive a higher quality of healthcare services [6]. Sanitation facilities are also better in urban than rural areas [3,5]. Like the urban-rural disparity, the rich-poor disparity is also obvious throughout the country as most public policies are urban-oriented [1,3,8,11-18].

Health disparities can be studied by social class, gender, ethnicity and rural-urban location [19,20]. Any kind of disparity is a matter of social injustice, which can increase the health risks for the disadvantaged population, weaken the pace of overall development and affect population health [8,12-14,17,21-24]. Therefore, reducing the disparity between different groups is an important component of the development of the country [15] as well as a key strategy to combat poverty and improve public health [25]. However, achieving equity in health and development may not be easy without adequate measures for the disadvantaged areas and groups of people [13].

The major objectives of this study were to demonstrate the trends and disparities in various public-health-related indicators (considered as dependent variables) by two equity markers in Bangladesh; namely, rural versus urban location and richest versus poorest quintiles of wealth index. In order to do so, first we presented our findings using figures to show (i) whether these indicators followed similar trends (e.g. increasing or decreasing) for both markers during 1993-2011 and (ii) whether the disparities were still significant after controlling for some important socioeconomic factors. To fulfill the second objective, we only used the most recent data, from the Bangladesh Demographic and Health Survey (BDHS) conducted in 2011. Such group-specific analyses might be important to monitor, for example, the overall performance of Bangladesh towards achieving the millennium development goals. They could also provide better information for policymakers and stakeholders because aggregated analyses have the potential to hide persistent differences between different groups [3]. This study could also be instrumental in identifying indicators that need more attention to enhance the progress of the country towards the millennium development goals. It should be noted that, in the absence of reliable data on income, an assets-based wealth index is a widely used proxy for the economic status of households [18]. Women were chosen for the analyses because they are more vulnerable in terms of poverty, illiteracy, discrimination, low empowerment and higher levels of reproductive health problems [3,21,23,26,27].

## Methods

### Sources of data

This study is based on the extensive analysis of six comparable data sets from the BDHS carried out in 1993-94, 1996-97, 1999-2000, 2004, 2007 and 2011. Detailed descriptions of the study designs, including informed consent and data collection, were explained in the country-specific reports [28]. All the Demographic and Health Surveys (DHS) were nationally representative and employed a common methodology across participating countries [29]. Many developing countries of the world routinely conduct similar surveys under the DHS programme. All these surveys were financially and technically supported by the United States Agency for International Development (USAID) [28]. The authors received all the data sets from MEASURE DHS. The DHS data collection procedures were ethically approved by the ORC Macro (Calverton, Maryland) Institutional Review Board [30]. Moreover, all these surveys were approved by the relevant authority of the Ministry of Health and Family Welfare in Bangladesh. A group of trained interviewers conducted face-to-face interviews for data collection. Before starting each interview, the interviewers also explained the objectives of the survey and received informed consent from the respondents.

### Sampling and sample sizes

Using multistage stratified cluster sampling, a representative sample of women, normally aged 15 to 49 years, was identified for each survey and then data was collected using a pre-tested questionnaire. Different sample sizes were used in different surveys and ranged from 9,127 in 1996-1997 to 11,440 in 2004. The overall response rate was very high for each survey, with a minimum rate of 96.7% [5,31-35]. In this study, 604 women aged 10-14 years were excluded from the analyses. After exclusion, the sample sizes were 9,493 (1993-94), 8,991 (1996-97), 10,373 (2000), 11,300 (2004), 10,996 (2007) and 17,749 (2011).

### Selected indicators as dependent variables and their public health relevance

A total of six public-health-related indicators were considered as dependent variables. Each of these indicators, including their categories and public health relevance, is explained below.

(i) The first indicator is ‘age at marriage in years (AAM)’. This was dichotomised using a cut-off point of 18 years, where AAM=0 if the marriage occurred before the age of 18 years, and AAM=1 if the marriage occurred at the age of 18 years or later. Marriage below the age of 18 years (i.e. early marriage) is negatively associated with education and positively associated with reproductive health problems such as non-contraception and high fertility, early, unplanned and unwanted pregnancies, shorter birth spacing and an increased risk of maternal and infant morbidity and mortality including sexually transmitted diseases [36-39].

(ii) The second indicator is the ‘ideal number of children (INC)’, where INC > 3 was coded as 0 and INC ≤ 2 was coded as 1. This is an important indicator of future fertility preferences, which reflects the total number of children a woman or man would wish for if she or he could start afresh. It can provide information about the excess of past fertility over ideal family size, which is a measure of unwanted fertility [5]. It can also be used to guess the fertility norms and levels of a population if women’s preferences prevailed [40].

(iii) The third indicator - adequate antenatal care (AANC) - was based on the number of visits that a woman completed during her most recent pregnancy. Maternal mortality is very high in Bangladesh and mostly (about 85%) associated with direct obstetric complications [16,41]. Generally antenatal care (ANC) is an important strategy for safe delivery and to reduce maternal mortality. It can also improve the health of women and their babies because they may receive necessary health information and services during consultations or check-ups [42-44]. Following a similar cut-off point used by other studies [42,44-46], four or more ANC visits were termed adequate (coded as 1), whereas less than four visits were considered inadequate (coded as 0).

(iv) The fourth indicator - delivery of the most recent child at any healthcare facility (DHF) - was dichotomised as yes or no. Here no means home delivery, which generally occurs in the absence of skilled professional attendants. Deliveries assisted by skilled professionals (such as doctors and nurses working at a healthcare facility) are normally safe and can reduce both maternal mortality and morbidity, which ultimately helps to make progress towards the Millennium Development Goal of improving maternal health [11,45]. Since Bangladesh is still one of the highest maternal mortality countries in the world, deliveries at healthcare facilities are imperative to reduce maternal mortality by managing emergency obstetric conditions such as excessive bleeding and obstructed labours requiring an operation promptly [11,47].

(v and vi) The fifth and sixth indicators, namely ‘being underweight’ and ‘being overweight’ among women are related to extreme body mass index (BMI). A woman was underweight when her BMI < 18.5 kg/m^2^ (coded as 1) and overweight when her BMI ≥ 25 kg/m^2^ (coded as 2). An intermediate BMI (18.5 to 25.0 kg/m^2^) was considered normal (coded as 0). Both extremes are reported to be associated with a variety of complications. Some of the adverse health outcomes of underweight are preterm births, low birth-weight babies, poor psychological health and high mortality. Similarly, overweight women are associated with lifestyle and non-communicable diseases such as diabetes, cardiovascular diseases, high cholesterol and hypertension [27]. Paradoxically, the co-existence of both extremes in Bangladesh indicates a dual burden of malnutrition, which needs group-specific attentions [27,30].

The sample sizes used in this study varied from indicator to indicator. We used the total sample for AAM and INC. For AANC and DHF, we used the sub-sample who gave birth during a defined period preceding the survey (three years for the survey of 1993-94 and five years for the others). No data was available in the BDHS 1993-94 for the underweight and overweight indicators. All indicators were dichotomised except being underweight and overweight.

### Statistical analysis

**Figure 1:**
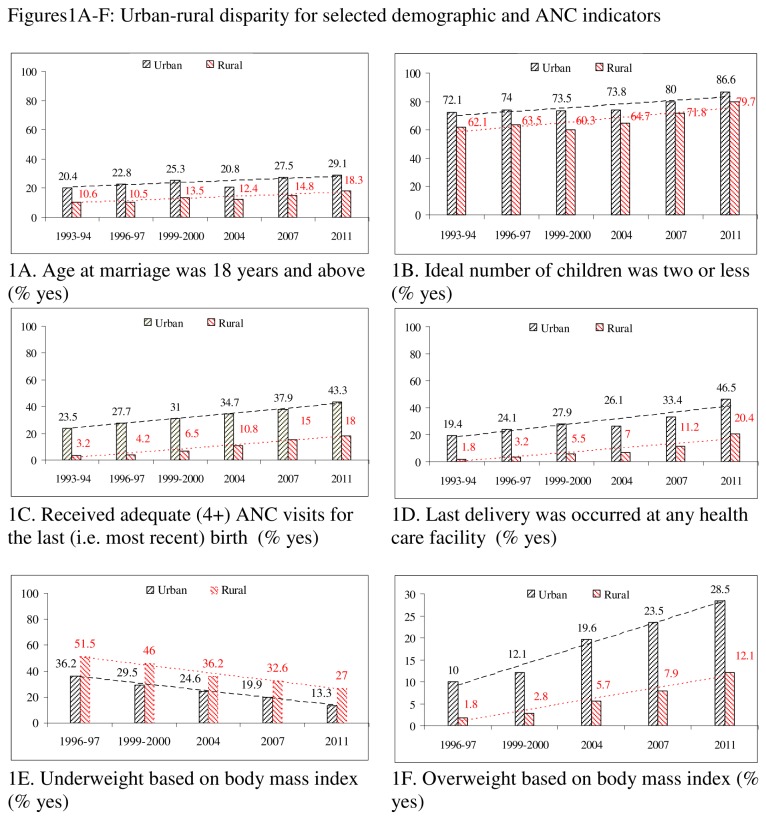
A-F: Urban-rural disparity for selected demographic and ANC indicators.

**Figure 2:**
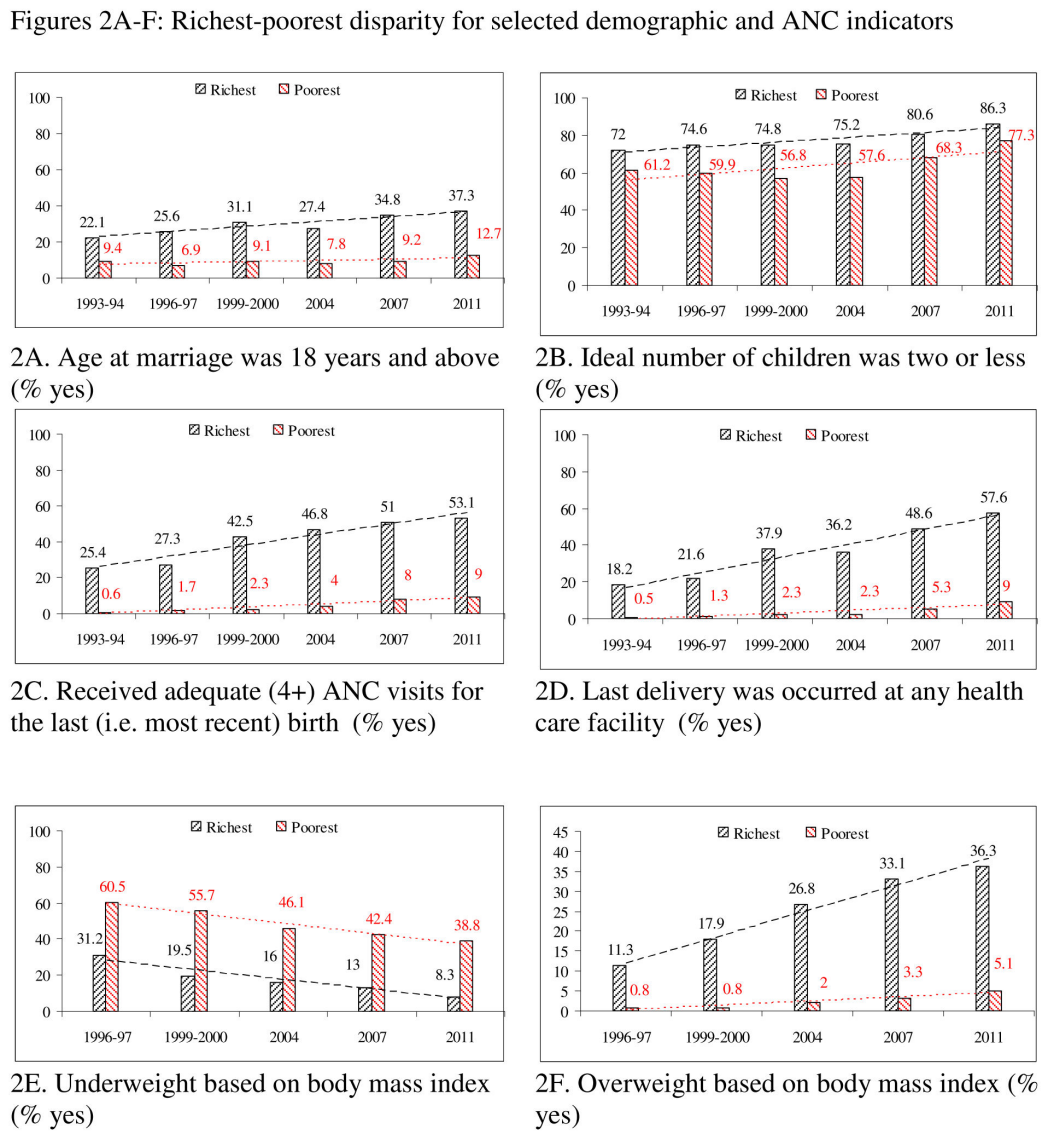
A-F: Richest-poorest disparity for selected demographic and ANC indicators.

IBM SPSS 20.0 was used to perform the statistical analyses. The percentages of the category coded as either 1 or 2 were calculated by SPSS and then used in Microsoft Office Excel 2003 to generate figures (Figures 1 and 2). These figures were used to show not only levels but also the linear trend lines (dotted) by rural-urban location (Figure 1) and richest-poorest groups (Figure 2). Using the most recent data, from the BDHS 2011, the comparative levels for the different indicators were reported to show the magnitude of disparities. Urban/rural and richest/poorest ratios for these indicators were also calculated. In general, a ratio equal to one indicates no disparity, which means the greater the deviation from the ratio one, the greater is the disparity. For all the indicators except being overweight, urban/rural and richest/poorest ratios greater than one indicate a better public health situation in the urban and richest groups as compared to their counterparts. In contrast, for the overweight indicator a ratio greater than one indicates a worse situation in the urban and richest groups.

Finally, multiple (either binary or multinomial) logistic regression analyses (based on the BDHS 2011) were performed depending on the categories of the indicators. For the dichotomous indicators, we performed a multiple binary logistic regression and for the extreme BMI indicators (with three categories), we applied a multiple multinomial logistic regression, taking the normal group as the reference category. We divided our findings into two models (Model I and II). Model I was used to study the urban-rural disparity, which was adjusted for several potential variables, namely: age, education, region of residence, frequency of watching television, sex of the head of household (except for the indicator ANNC), and type of toilet facility. For this model, we presented findings, namely: odds ratios (ORs) and a 95% confidence interval (95% CI) for all the model variables. Significance levels were also reported by asterisks. We selected adjusted variables based on their significant associations with indicators [11,16,18,27,37,44] and our findings from the bivariable analyses. Then we applied Model II to estimate the ORs and 95% CI for the richest group (taking the poorest as reference category) for the indicators, where the model was adjusted for age, urban-rural place of residence, region of residence, frequency of watching television, and sex of the head of household. Education and type of toilet facility were dropped from Model II to reduce the problem of multicollinearity with wealth index. To show the goodness of fit statistics, we reported the Nagelkerke R^2^ and overall classification percentage.

## Results

### Trend analysis for five different health surveys

All indicators except being overweight (1993-2011) revealed gradual improvements in both urban and rural areas (Figure 1). For instance, an increasing trend was observed for the indicator AAM (above 18 years), which increased from 10.6% in 1993-94 to 18.3% in 2011 in rural areas and from 20.4% to 29.1% in urban areas. Adequate ANC (4+ visits) and delivery at a healthcare facility also increased in both areas. The prevalence of underweight individuals decreased from 51.5% to 27.0% in rural area and from 36.2% to 13.3% in urban areas during 1993-2011. Although the prevalence of overweight individuals increased in both urban and rural locations, the pace was faster in urban areas than in rural ones. Hence the urban-rural gap with respect to overweight individuals is increasing.

Like the changes in urban-rural locations, we also observed positive changes among the richest and poorest groups for all the indicators except being overweight (Figure 2). However, the rate of change was faster in the richest group as compared to the poorest one. As a result, disparities are increasing for most of the indicators, namely: AAM, AANC, DHF, and being overweight. For the other indicators (INC and being underweight), disparities over time remained almost the same.

### Rural-urban and richest-poorest comparison based on BDHS 2011

The levels of the different indicators differed significantly between urban-rural and richest-poorest groups (Table 1). For instance, urban pregnant women received adequate ANC services more often (43.3%) than rural women (18.0%). The same indicator also differed significantly between the richest (53.1%) and poorest (9.0%) groups. At a glance, significantly lower levels of AAM, INC, AANC, and DHF and higher levels of being underweight in the rural and poorest groups revealed their greater level of vulnerability than the urban and richest groups. The rural and poorest groups only revealed a better situation in terms of being overweight, because it was significantly lower in these groups.

**Table 1:** Comparison of different indicators (i.e. dependent variables) by spatial (rural versus urban) and social (poorest versus richest) groups based on BDHS 2011.

Indicators		Urban (%)	Rural (%)	Urban/rural ratio	P	Richest	Poorest	Richest/poorest ratio	P
Age at first marriage: ≥ 18 years		29.1	18.3	1.59	< 0.001	37.3	12.7	2.93^***^	< 0.001
Ideal number of children: ≤ 2 children		86.6	79.7	1.09	< 0.001	86.3	77.3	1.12^***^	< 0.001
Received adequate (≥ 4) ANC visits for the most recent child: yes		43.3	18.0	2.41	< 0.001	53.1	9.0	5.90^***^	< 0.001
Delivered most recent child at any healthcare facility: yes		46.5	20.4	2.28	< 0.001	57.6	9.0	6.40^***^	< 0.001
Underweight (BMI < 18.5 kg/m^2^): yes		13.3	27.0	0.49	< 0.001	8.3	38.8	0.21^***^	< 0.001
Overweight (BMI ≥ 25.0 kg/m^2^): yes		28.5	12.1	2.34	< 0.001	36.3	5.1	7.12^***^	< 0.001

^***^ P < 0.001, ^*^P < 0.05 (based on Chi-square test for equality of proportions)

Based on the urban/rural ratio, higher disparities (deviation from 1.0) were found for AANC (ratio = 2.41), being underweight (ratio = 2.03), DHF (ratio = 2.28) and AAM (ratio = 1.59). For the overweight indicator the ratio was 0.49, which also indicated greater vulnerability in urban areas. Comparatively, disparities were stronger between the richest-poorest than the urban-rural groups. Disparities for the INC indicator were lowest for both equity markers.

### Bivariate analyses between indicators and adjusted variables

Bivariate analyses between adjusted variables and indicators revealed significant associations (Table 2). The associations of different indicators (except being underweight) with education and frequency of watching TV were positive and significant. Sanitation facilities were significantly associated with all the indicators, with higher rates (except for underweight individuals) among the users of flush toilets. The underweight indicator revealed a negative association with education and frequency of watching TV. Although other variables, namely age, division, and sex of the head of household were significantly associated with the indicators, the results were mixed.

**Table 2:** Bivariate association of controlled variables with selected indicators based on BDHS 2011.

Variables	Categories	Age at first marriage in years		Ideal number of children		Received adequate (4+) ANC visits		Delivered most recent child at any healthcare facility		BMI (kg/m^2^)		
		≥ 18	< 18	≤ 2	2+	yes	no	yes	no	Underweight	Overweight	Normal
		%	%	%	%	%	%	%	%	%	%	%
Age in years	<25	19.1^***^	80.9	91.0^***^	9.0	25.3^***^	74.7	26.8^**^	73.2	28.5^***^	8.4^***^	63.2
	25-34	24.8	75.2	82.8	17.2	23.9	76.1	27.3	72.7	20.3	19.4	60.3
	35-49	19.3	80.7	71.9	28.1	16.1	83.9	21.2	78.8	22.2	20.4	57.5
Education in years	No	11.8^***^	88.2	71.7^***^	28.3	7.6^***^	92.4	10.0^***^	90.0	29.2^***^	10.9^***^	59.9
	1-5	13.9	86.1	78.9	21.1	15.1	84.9	16.3	83.7	26.0	13.4	60.5
	6-10	24.2	75.8	89.2	10.8	30.1	69.9	33.2	66.8	19.8	19.7	60.5
	11+	71.2	28.8	92.5	7.5	64.7	35.3	71.5	28.5	8.4	33.3	58.8
Division	Barisal	17.4^***^	82.6	81.0^***^	19.0	24.7^***^	75.3	19.1^***^	80.9	25.9^***^	12.6^***^	61.5
	Dhaka	27.0	73.0	70.7	29.3	18.7	81.3	22.9	77.1	21.5	17.6	60.8
	Chittagong	22.7	77.3	83.2	16.8	25.6	74.4	28.1	71.9	22.9	18.1	59.0
	Khulna	16.5	83.5	88.4	11.6	28.4	71.6	40.3	59.7	18.5	19.6	62.0
	Rajshahi	16.1	83.9	87.2	12.8	20.9	79.1	26.6	73.4	24.1	15.6	60.2
	Rangpur	13.0	87.0	85.5	14.5	33.5	66.5	23.9	76.1	26.3	10.6	63.0
	Sylhet	37.2	62.8	68.2	31.8	15.8	84.2	21.4	78.6	34.1	12.7	53.2
Frequency of watching TV	Not at all	15.5^***^	84.5	74.7^***^	25.3	12.3^***^	87.7	12.9^***^	87.1	30.8^***^	8.2^***^	61.0
	< 1 time/week	18.5	81.5	84.5	15.5	17.9	82.1	21.3	78.7	26.4	11.7	61.9
	≥ 1+ time/week	26.3	73.7	86.3	13.7	35.1	64.9	39.3	60.7	16.7	24.2	59.1
Household having electricity	No	13.5^***^	86.5	77.8^***^	22.2	13.3^***^	86.7	12.2^***^	87.8	32.4^***^	7.2^***^	60.4
	Yes	24.5	75.5	83.0	17.0	30.5	69.5	34.9	65.1	18.0	22.2	59.8
Sex of the household sex	Male	20.8^***^	79.2	81.9^***^	18.1	23.7	76.3	26.1^**^	73.9	23.3^***^	16.0^***^	60.7
	Female	24.1	75.9	78.2	21.8	25.6	74.4	31.5	68.5	24.5	19.8	55.6
Type of sanitation facility	Flush	34.2^***^	65.8	87.3^***^	12.7	50.3^***^	49.7	54.0^***^	46.0	9.3^***^	34.3^***^	56.4
	Pit latrine	18.2	81.8	80.4	19.6	20.2	79.8	22.7	77.3	24.7	14.0	61.3
	Others	12.4	87.6	74.9	25.1	10.7	89.2	9.2	90.8	38.0	4.5	57.4

Note: All variables are significantly associated with all indicators

^***^ P ≤ 0.001, ^**^P ≤ 0.01 (based on Chi-square test for independence)

### Multiple logistic regression analyses on the most recent survey (BDHS 2011)

Detailed results of the multiple binary logistic regression analyses (Model I) are presented in Table 3. As with the bivariable analyses, education and sanitation facilities consistently revealed strong associations with the indicators. For instance, the ORs for all the indicators (except the underweight category) were between two (for overweight individuals, OR = 2.13; 95% CI = 1.78–2.55) and 20 times (for AAM, OR=20.38; 95% CI = 17.10–24.29) greater among the higher secondary group than the group with no education. Frequency of watching TV also indicated results consistent with the bivariable analyses, although this variable became insignificant for the AAM indicator. Divisional disparities were significant for all indicators but the results were mixed. Age remained significant for all of the indicators except DHF. Finally, sex of the head of household revealed a significant association with three indicators only.

**Table 3:** Multiple analyses for selected indicators based on BDHS 2011.

Variables	Categories	Binary logistic regression analyses				Multinomial logistic regression analysis	
		Age at first marriage: ≥ 18 years	Ideal number of children: ≤ 2 children	Received adequate (4+) ANC visits: yes	Delivered most recent child at health facility: yes	Under-weight (BMI < 18.5 kg/m^2^): yes	Over-weight (BMI ≥ 25.0 kg/m^2^): yes
		OR (95% CI)	OR (95% CI)	OR (95% CI)	OR (95% CI)	OR (95% CI)	OR (95% CI)
**Model I**		(N=16,441)	(N = 16,235)	(N = 6,587)	(N = 6,593)	(N = 16,072)	(N = 16,072)
Age in years	<25	1.00	1.00	1.00	1.00	1.00	1.00
	25-34	1.58 (1.42-1.76)^***^	0.52 (0.46-0.59)^***^	0.94 (0.82-1.07)	1.05 (0.92-1.20)	0.65 (0.59-0.71)^***^	2.68 (2.35-3.06)^***^
	35-49	1.65 (1.47-1.86)^***^	0.31 (0.27-0.35)^***^	0.73 (0.55-0.96)^*^	1.08 (0.84-1.40)	0.66 (0.60-0.74)^***^	3.62 (3.16-4.15)^***^
Education	No	1.00	1.00	1.00	1.00	1.00	1.00
	Primary	1.30 (1.15-1.74)^***^	1.12 (1.01-1.24)^*^	1.77 (1.38-2.26)^***^	1.43 (1.14-1.80)^***^	0.89 (0.80-0.98)^*^	1.32 (2.35-3.06)^***^
	Secondary	2.83 (2.50-3.21)^***^	1.95 (1.72-2.20)^***^	3.62 (2.85-4.58)^***^	3.07 (2.46-3.82)^***^	0.71 (0.64-0.80)^***^	1.88 (1.64-2.15)^***^
	Higher secondary	20.38 (17.10-24.29)^***^	2.70 (2.12-3.44)^***^	12.05 (8.95-16.23)^***^	10.99 (8.24-14.66)^***^	0.41 (0.32-0.52)^***^	2.13 (1.78-2.55)^***^
Division	Barisal	1.00	1.00	1.00	1.00	1.00	1.00
	Chittagong	2.02 (1.64-2.50)^***^	0.48 (0.40-0.59)^***^	0.53 (0.40-0.72)^***^	1.05 (0.76-1.44)	0.87 (0.72-1.05)	1.30 (1.02-1.66)^*^
	Dhaka	1.43 (1.16-1.76)^***^	1.06 (0.88-1.28)	0.62 (0.47-0.83)^***^	1.12 (0.82-1.53)	1.05 (0.89-1.26)	1.08 (0.85-1.36)
	Khulna	0.94 (0.75-1.19)	1.82 (1.46-2.27)^***^	0.83 (0.60-1.15)	2.37 (1.69-3.32)^***^	0.75 (0.62-0.92)^**^	1.46 (1.14-1.87)^**^
	Rajshahi	1.04 (0.83-1.31)	1.70 (1.38-2.09)^***^	0.79 (0.58-1.08)	1.58 (1.13-2.21)^**^	0.93 (0.77-1.12)	1.26 (0.98-1.61)
	Rangpur	0.85 (0.67-1.08)	1.54 (1.24-1.91)^***^	1.79 (1.31-2.43)^***^	1.58 (1.12-2.23)^**^	0.91 (0.75-1.10)	0.98 (0.75-1.28)
	Sylhet	4.11 (3.24-5.23)^***^	0.52 (0.42-0.66)^***^	0.57 (0.39-0.83)^**^	1.33 (0.90-1.95)	1.47 (1.18-1.82)^***^	1.09 (0.80-1.49)
Frequency of watching TV	Not at all	1.00	1.00	1.00	1.00	1.00	1.00
	< 1 time/week	1.00 (0.87-1.16)	1.54 (1.34-1.77)^***^	1.04 (0.83-1.31)	1.39 (1.12-1.73)^**^	0.90 (0.80-1.02)	1.25 (1.05-1.48)^*^
	≥ 1+ time/week	1.01 (0.91-1.12)	1.43 (1.30-1.59)^***^	1.80 (1.54-2.11)^***^	2.10 (1.80-2.45)^***^	0.73 (0.66-0.80)^***^	2.02 (1.79-2.28)^***^
Sex of the household head	Male	1.00	1.00	-	1.00	1.00	1.00
	Female	0.99 (0.86-1.13)	0.97 (0.85-1.10)		1.31 (1.05-1.63)^*^	1.23 (1.08-1.40)^**^	1.20 (1.04-1.39)^*^
Type of sanitation facility	Flush	1.00	1.00	1.00	1.00	1.00	1.00
	Pit latrine	0.85 (0.75-0.96)^**^	0.85 (0.73-0.98)^*^	0.50 (0.42-0.60)^***^	0.53 (0.45-0.64)^***^	1.77 (1.51-2.07)^***^	0.65 (0.58-0.74)^***^
	Others	0.82 (0.67-0.99)^*^	0.71 (0.59-0.86)^***^	0.42 (0.32-0.57)^***^	0.35 (0.26-0.48)^***^	2.36 (1.95-2.85)^***^	0.34 (0.26-0.44)^***^
Place of residence	Rural	1.00	1.00	1.00	1.00	1.00	1.00
	Urban	1.12 (1.01-1.25)^*^	1.23 (1.08-1.39)^***^	2.10 (1.79-2.46)^***^	1.95 (1.67-2.28)^***^	0.75 (0.67-0.84)^***^	1.47 (1.32-1.65)^***^
Model I summary	Nagelkerke R^2^	0.21	0.16	0.26	0.27	0.17^a^	0.17^a^
	Overall classification (%)	82.6	81.1	79.7	78.1	60.6	60.6
**Model II**		N = 17,745	N = 17,535	N = 7,306	N = 7,312	N = 17,306	N = 17,306
Wealth Index	Poorest	1.00	1.00	1.00	1.00	1.00	1.00
	Richest	3.72 (3.20-4.34)^***^	1.32 (1.12-1.56)^***^	8.35 (6.52-10.70)^***^	8.47 (6.66-10.76)^***^	0.26 (0.22-0.30)^***^	4.88 (3.98-5.99)^***^
Model II summary	Nagelkerke R^2^	0.10	0.14	0.23	0.24	0.18^a^	0.18^a^
	Overall classification (%)	79.0	81.5	77.9	76.7	60.6	60.6

^***^ P ≤ 0.001, ^**^P ≤ 0.01, ^*^P ≤ 0.05; ^a^ Pseudo Nagelkerke R^2^

Model II: Adjusted for age, place of residence, division, frequency of watching TV and sex of the household head

Disparities by two equity markers remained significant even in the multiple analyses. For instance, the INC (≤ 2 children) was 1.23 times higher (OR=1.23; 95% CI = 1.08–1.39) in urban areas than in rural areas. Significantly higher likelihoods in urban areas were also found for AANC (OR = 2.11; 95% CI = 1.80–2.47) and DHF (OR = 1.95; 95% CI = 1.67–2.28) and being overweight (OR = 1.47; 95% CI = 1.32–1.65). The likelihood of being underweight was 25% lower in urban areas (OR = 0.75; 95% CI = 0.67–0.84) than in rural areas. However, richest-poorest disparities were more pronounced than urban-rural disparities. For example, the likelihood for AANC was 2.11 for the urban-rural disparity, whereas it was 8.34 for the richest-poorest disparity. The likelihood of being underweight was 0.75 for the urban-rural disparity, while it was 0.26 for the richest-poorest disparity.

## Discussion

Our study presents long-term trends for some public-health-related indicators in Bangladesh based on representative data. It extracted several interesting findings that are important for policymakers and stakeholders. One of the important findings is the increasing tendency of all indicators (except being underweight) over time, which delivers the message that both urban and rural areas are progressing (but not in the sense of being overweight) in Bangladesh. However, urban-rural gaps for all indicators seem to be similar except for the increasing gap in being overweight. Some of the driving forces behind such development are already mentioned in the introduction. Unfortunately, our findings from the perspective of the richest and poorest groups are disappointing, because richest-poorest disparities are widening for some of these indicators. These findings are not consistent with the urban-rural disparities, which are mostly stable. These results may imply that, although overall Bangladesh is progressing due to the many interventions, these were not as effective for the poorest segments of society. The third important finding is the increasing proportion of overweight individuals in the urban and richest groups, who are more educated. To control this emerging problem, increasing efforts are needed to target them. It is also important to find ways to keep the prevalence of being overweight lower in rural areas.

In the next few paragraphs we attempt to discuss the implications of our findings. It is clear that rural areas are far behind in terms of both positive (AAM, AANC, DHF, INC) and negative (underweight) indicators. Traditionally, rural areas of Bangladesh are relatively underserved by the relevant authorities. Although many rural-based governmental and non-governmental development organisations are implementing various promotional activities for the underserved areas (e.g. focusing on poverty, literacy and healthcare services), these might not be adequate to minimise the existing urban-rural gaps. Therefore, rural areas need more efforts and activities from policymakers and stakeholders to reduce the urban-rural gaps. Rural women should have more participation in political and decision-making processes [48] including educational institutions and health services [49]. Particular attention should be given to the reproductive-health-related millennium development indicators in Bangladesh, which are far below the aimed-at level in both urban and rural areas. It has already been mentioned that bleeding and the unsafe termination of pregnancies are the main direct causes of maternal deaths in Bangladesh. Some of the indirect causes are limited availability and affordability of professional care, the long distances to health centres in rural areas and the lack of trained medical personnel [50]. In rural Bangladesh, the malnourishment rate among women is one of the highest in the world [51] and poverty is the strongest predictor for this [52,53], although many interventions like the Essential Service Package are implemented by the government [54,55]. Another challenge is the lack of trained community-based skilled birth attendants and general health personnel, who provide antenatal care and delivery assistance [56].

According to Westoff and Koffman, ‘television exposes viewers to aspects of modern life that compete with traditional attitudes toward marriage and the family and lead to views and behavior conducive to the control of fertility’ [57]. Mass media can disseminate information about the consequences of unsafe and complicated deliveries [11]. Since the rural and poorest groups have much more limited access to mass media (e.g. television) than their urban and richest counterparts, increasing access to television for these disadvantaged groups could be another important strategy to narrow down the disparities between health outcomes. Here it should be mentioned that weekly access to TV in rural areas increased from 11.7% to 38.0% during 1993-2011, although these rates were far below those of urban areas (18.5% in 1993-94 and 77.9% in 2011) (data not shown). Like mass media, the lack of appropriate transportation in rural areas puts them in a disadvantaged position to use basic services (e.g. healthcare). Therefore, feasible strategies to reduce transportation and other accessibility barriers for improving the care-seeking behaviour among poor and rural residents are necessary [54,55].

Reducing rich-poor and urban-rural health inequalities is also imperative for public health and sustainable development. Our future strategies should be based on the principles of equity and quality, not on the urban locality and economic capacity of the population. Some of the approaches are: (i) focusing on the poorest groups through specific interventions; (ii) setting reasonable targets to improve their health; (iii) providing healthcare services according to people’s needs irrespective of socioeconomic status; (iv) fostering and expanding public–private partnerships, mainly in rural areas; (v) developing and strengthening the public health infrastructure and (vi) strategies to effectively address accessibility barriers [3,11,16,18,37]. Health-related policy should incorporate strategies for increasing women’s level of education, economic status and decision-making power to improve maternal healthcare and survival [11,52]. The public health system must be equipped to provide emergency services during pregnancy and delivery to encourage poor and deprived women [16]. Better coordination among all health-related stakeholders to jointly develop comprehensive strategies to promote ANC and expansion of the existing financing programme and the Maternal Health Voucher Scheme are also important. As women feel more comfortable consulting female healthcare providers because of patriarchal society and Islamic values, more female providers should be recruited and trained in all parts of Bangladesh. The last but equally important step is to increase awareness levels among women seeking ANC services in time [11].

What can be done to reduce the rising prevalence of overweight individuals in Bangladesh? As both underweight and overweight individuals exist in close proximity in Bangladesh [30], simultaneous applications of public health interventions might not be so simple. Because if any intervention is targeted at preventing one problem, it might exacerbate the other. For instance, a dietary recommendation to consume reduced levels of fat at the family level may reduce the prevalence of being overweight, but this may accelerate the risk of being underweight for other members of the same family. In this situation, health education interventions specific to the nutritional problem may be optimal. For instance, overweight people should be advised to consume fewer calories and to eat healthy diets (e.g. increasing their consumption of fruits and vegetables). More physical activity, walking, and doing sports should be recommended for them. Other programmes such as providing incentives for food-producing institutions to produce less fatty foods, controlling the nutritional status from childhood, and systematic monitoring and surveillance of nutritional status are also important to address the widespread problem of being underweight or overweight [27].

As early marriage is a human-rights violation and is associated with many negative consequences, the first and foremost strategy to prevent early marriage should be the strict enforcement of existing laws and policies. Creating job opportunities for rural women and poor and changing cultural perceptions to ensure that early marriage is no longer an economically feasible and socially acceptable option for impoverished families are important. Moreover, increasing access to and support for contraceptive methods is urgently needed for young married women, their spouses and families to reduce the high fertility and poor fertility-control outcomes of this practice [37].

### Limitations and strengths of the study

Our study is not free from limitations. One of these is information bias, which may result from e.g. self-reporting age, age at marriage, and education. BMI is a crude index which does not consider the distribution of fat and which can vary from individual to individual. This study used cross-sectional data which could not confirm the cause and effect relationships. The results may be biased as some variables, such as smoking, physical activity and dietary habits, are not adjusted for underweight and overweight indicators in the multinomial logistic regression [27]. Although propensity score matching and inverse weighted estimator are two possible solutions to reduce selection bias, we did not apply them keeping in mind that many authors frequently applied multiple logistic regressions in similar studies. Generally multiple logistic regressions are easier to conduct than propensity score matching for addressing several outcome variables. The ideal number of children could be related to the number of children actually born, because women with more children may report a greater number of children as ideal to justify their fertility [58]. Moreover, all the indicators should be critically checked for intra-urban disparity (e.g. slum versus non-slum), since the rural-urban disparity remained almost the same over a long period. Unfortunately, relevant data were not available to enable an analysis of the slum versus non-slum disparity. Perhaps the increasing slum population in urban areas is a factor in this regard. It is likely that the overall impact of interventions in urban areas is underestimated for the non-slum population and overestimated for the slum population. Although divisional disparities are found, these are not focused like the other two equity indicators. It should also be mentioned that Bangladesh is a country with huge population growth. Generally trends are valid provided the population growths are more or less constant for different sectors with appropriate sampling weights for all surveys. Briefly, the strengths of the study are related to the use of several indicators based on multiple data sets. This gives an idea of existing disparities, which should be urgently addressed.

## Conclusions

Our study demonstrated that there have been positive changes in health indicators in both urban and rural areas. However, the rural and poorest groups are still more vulnerable in terms of health indicators (except being overweight) compared to the urban and richest groups. Although positive changes are found in both the richest and poorest groups, unfortunately richest-poorest disparities are widening. Based on our results, we underscore the necessity for more interventions for rural areas in general and for the poorest group in particular. Problem-specific interventions are clearly needed to address the dual burden of underweight and overweight individuals. Health education should be an important strategy in this regard. All the policies and interventions should be guided by the principles of equity and quality irrespective of social status. Therefore, new and existing programmes should be further developed and extended to reach more women living in rural Bangladesh.
